# Anti-Inflammatory Cytokine Interleukin-4 Inhibits Inducible Nitric Oxide Synthase Gene Expression in the Mouse Macrophage Cell Line RAW264.7 through the Repression of Octamer-Dependent Transcription

**DOI:** 10.1155/2013/369693

**Published:** 2013-12-29

**Authors:** Miki Hiroi, Yoshiichi Sakaeda, Hana Yamaguchi, Yoshihiro Ohmori

**Affiliations:** Division of Microbiology and Immunology, Department of Oral Biology and Tissue Engineering, Meikai University School of Dentistry, 1-1 Keyakidai, Sakado 350-0283, Saitama, Japan

## Abstract

Inducible nitric oxide synthase (iNOS) is a signature molecule involved in the classical activation of M1 macrophages and is induced by the *Nos2* gene upon stimulation with Th1-cell derived interferon-gamma (IFN**γ**) and bacterial lipopolysaccharide (LPS). Although the anti-inflammatory cytokine IL-4 is known to inhibit *Nos2* gene expression, the molecular mechanism involved in the negative regulation of *Nos2* by IL-4 remains to be fully elucidated. In the present study, we investigated the mechanism of IL-4-mediated *Nos2* transcriptional repression in the mouse macrophage-like cell line RAW264.7. Signal transducer and activator of transcription 6 (Stat6) knockdown by siRNA abolished the IL-4-mediated inhibition of *Nos2* induced by IFN**γ**/LPS. Transient transfection of a luciferase reporter gene containing the 5′-flanking region of the *Nos2* gene demonstrated that an octamer transcription factor (OCT) binding site in the promoter region is required for both positive regulation by IFN**γ**/LPS and negative regulation by IL-4. Although IL-4 had no inhibitory effect on the DNA-binding activity of constitutively expressed Oct-1, IL-4-induced *Nos2*-reporter transcriptional repression was partially attenuated by overexpression of the coactivator CREB-binding protein (CBP). These results suggest that a coactivator/cofactor that functionally interacts with Oct-1 is a molecular target for the IL-4-mediated inhibition of *Nos2* and that IL-4-activated Stat6 represses Oct-1-dependent transcription by competing with this coactivator/cofactor.

## 1. Introduction

Macrophages function in various aspects of the inflammatory reaction, innate and acquired immunity, and tissue remodeling, and the functional competence of macrophages are generally acquired in response to a diverse array of stimuli encountered in the tissue microenvironment [1]. Bacterial cellular components, such as lipopolysaccharide (LPS) and type I helper T cell (Th1)-derived cytokine interferon-gamma (IFN*γ*), are well-known macrophage-activating stimuli that promote antimicrobial and antitumor functions [1–3]. These so-called classically activated macrophages, or M1 macrophages, produce large amounts of proinflammatory cytokines, reactive oxygen intermediates, and such reactive nitrogen intermediates as nitric oxide (NO), which is generated by inducible NO synthase (iNOS) encoded by the *Nos2* gene [4, 5]. In contrast, the Th2-derived cytokines interleukin-4 (IL-4) and IL-13 induce an alternative mode of macrophage activation, resulting in macrophages that participate in scavenging, the anti-inflammatory response, wound healing, and tissue remodeling by enhancing the expression of the mannose receptor, the IL-1 receptor antagonist, and arginase I [3, 6–8]. These Th2-derived cytokines also inhibit the expression of proinflammatory genes, including *Nos2*, in classically activated macrophages [9–12], thereby further promoting the polarization toward the type II response.

The intracellular signaling pathway for IL-4 is, at least in part, mediated by signal transducer and activation of transcription 6 (Stat6), a latent cytoplasmic transcription factor that is phosphorylated at a tyrosine residue (Tyr641) by Janus kinase 1 (Jak1) after IL-4 binds to the IL-4 receptor [13, 14]. Phosphorylated Stat6 assembles in a dimeric form, translocates to the nucleus, binds to a specific *cis*-regulatory sequence, and mediates the transcriptional activation of IL-4-inducible genes [13–18]. IL-4-induced Stat6 also functions as a negative regulator of the IFN*γ*-induced Stat1-dependent transcriptional activation of macrophage genes, and previous studies have shown that Stat6 directly and/or indirectly suppresses IFN*γ*-induced Stat1-dependent transcription [19–22]. Although IL-4-activated Stat6 appears to be indispensable for the negative regulation of IL-4 [17, 19], the molecular mechanisms by which IL-4-activated Stat6 inhibits the macrophage gene expression induced by LPS and IFN*γ* remain to be fully clarified.

Transcriptional regulation of the mouse *Nos2* gene induced by LPS and IFN*γ* in macrophages has been extensively studied [23–30]. The 5′-proximal region of the *Nos2* gene (region I) contains a TATA box and binding motifs for octamer transcription factor (OCT) and nuclear factor *κ*B (NF-*κ*B), which primarily mediate the transcriptional activation induced by LPS [23, 24, 31–33]. The distal regulatory region (region II) located 0.9 kb upstream from the transcription start site principally contains regulatory sequences for mediating IFN-induced activation. In this region, IFN-stimulated response elements (ISREs) and the IFN*γ*-activation sequence (GAS) have been identified as binding sties for members of the IFN regulatory factor (IRF) family, IFN-stimulated gene factor 3 (ISGF3 composed of Stat1, Stat2, and IRF-9), and Stat1 [25–28, 30], which mediate responsiveness to IFNs. The transcriptional synergy of the *Nos2* gene induced by IFN*γ* and LPS has been suggested to be mediated by a functional cooperation between LPS-activated transcription factors, such as NF-*κ*B, at region I and IFN-induced transcription factors at region II [23, 26, 30]. IL-4 also negatively regulates the expression of the *Nos2* gene, particularly the IFN*γ*-induced expression of *Nos2* in mouse macrophages [12]. The mechanisms involved in the IL-4-mediated inhibition of *Nos2* have been shown to depend upon the negative regulation of IFN*γ*-induced IRF-1 through the inhibition of IRF-1 expression [34, 35], through competition for IRF-1 binding with IL-4-induced IRF-2 [36], or through an attenuation of the interaction with IFN consensus sequence-binding protein (ICSBP, known as IRF-8) [28]. Although the mechanisms involved in the IFN*γ*-induced expression of the *Nos2* gene by IL-4 have focused on the negative regulation of IRF-1, the mechanism of the IL-4-mediated inhibition of the *Nos2* gene induced by IFN*γ* and LPS remains to be fully elucidated.

In the present study, we analyzed the molecular mechanisms by which IL-4 inhibits the transcriptional activation of the mouse *Nos2* gene upon stimulation with IFN*γ* and LPS in the mouse macrophage cell line RAW264.7. We demonstrated that Stat6 knockdown by siRNA abolishes the IL-4-mediated inhibition of *Nos2* mRNA expression. Using the transient transfection of a luciferase reporter gene containing the 5′-regulatory region of the *Nos2* gene, we identified the OCT site in the proximal promoter region of the *Nos2* gene as the responsive region for IL-4-mediated repression. These data indicate that IL-4-activated Stat6 inhibits the OCT-dependent transcriptional activation of the *Nos2* gene in RAW264.7 cells.

## 2. Materials and Methods

### 2.1. Reagents

LPS prepared using Westphal phenolic extraction from *Escherichia coli* (0111:B4) was obtained from Sigma-Aldrich Corporation (St. Louis, MO, USA). Recombinant mouse IFN*γ* and IL-4 were obtained from Chemicon International (Temecula, CA, USA) and R&D Systems (McKinley Place, NE, USA), respectively. Rabbit polyclonal antibodies against Stat6 (sc-981), *β*-actin (sc-1616), Bob-1/BOB.1 (sc-955), TATA-binding protein (TBP; sc-204), Oct-1 (sc-232), and Oct-2 (sc-233) were obtained from Santa Cruz Biotechnology (Hercules, CA, USA).

### 2.2. Cell Culture

The mouse macrophage-like cell line RAW264.7 was obtained from American Type Culture Correction (Manassas, VA, USA) and cultured in Dulbecco's Modified Eagle's Medium (DMEM; Invitrogen, Grand Island, NY, USA) supplemented with 10% fetal bovine serum (FBS; Bio West, Miami, FL,USA) and 1% penicillin G/streptomycin sulfate (Invitrogen). For all experiments, the cells were subcultured until a 70%~80% confluent monolayer was achieved. The cells were then pretreated with 10 ng/mL recombinant mouse IL-4 for 30 min prior to stimulation with 10 ng/mL mouse IFN*γ* and/or 100 ng/mL LPS for the indicated time. The mouse B cell leukemia cell line BCL1-B20 (RCB2618) [37] was obtained from the RIKEN Bioresource Center (Tokyo, Japan) and cultured in RPMI1640 supplemented with 10% FBS and 1% penicillin G/streptomycin sulfate.

### 2.3. Determination of NO_2_^−^ Accumulation

Nitrite accumulation in the culture supernatant was measured by the Griess assay, as described previously [38]. Briefly, 100-*μ*L aliquots of culture supernatant were incubated with an equal volume of Griess reagent [1% sulfanilamide/0.1% *N*-(1-naphthyl) ethylenediamine dihydrochloride/2.5% H_3_PO_4_] at room temperature for 10 min. The absorbance of the samples at 550 nm was measured using a microplate reader (Thermo Fisher Scientific, Waltham, MA, USA), and the NO_2_^−^ content was determined using sodium nitrite as a standard. The cellular protein in each culture well was also determined by the Bradford method [39]. NO production was expressed as nmol per protein content.

### 2.4. Preparation of Total RNA and Northern Hybridization Analysis

The preparation of total RNA by the guanidine isothiocyanate-cesium chloride method and northern hybridization analysis were performed as previously described [40]. In some experiments, total RNA was prepared using the Fast Pure RNA kit (Takara, Otsu, Japan). The cDNA probes for mouse inducible nitric oxide synthase (*Nos2*) and rat glyceraldehyde-3-phosphate dehydrogenase (*Gapdh*) were described elsewhere [40, 41].

### 2.5. Quantitative Real-Time RT-PCR

cDNA was synthesized from the purified total RNA using a high-capacity cDNA reverse transcription kit (Life Technologies, Carlsbad, CA, USA) according to the manufacturer's instructions. Real-time PCR probes and primers specific for mouse *Nos2*, *Stat6*, *Oct-1*, and *Oct-2*, as shown in Table 1 in the Supplementary Material available at http://dx.doi.org/10.1155/2013/369693, were selected using the Universal Probe Library Assay Design Center (Roche Applied Science, Basel, Switzerland). Aliquots of cDNA were amplified using a LightCycler 480 Real-Time PCR System (Roche) and TaqMan Gene Expression Master Mix (Life Technologies) according to the manufacturer's instructions. The PCR cycling conditions were as follows: 95°C for 5 min and 40 cycles of 95°C for 10 s, 60°C for 30 s, and 72°C for 10 s. The transcript levels were calculated relative to the 18S rRNA levels as an internal control.

### 2.6. siRNA-Mediated Knockdown

Cells were seeded in 24-well plates and transfected with 100 nM of SMART pool siRNA targeting mouse Stat6 (a mixture of the four different Stat6 on-target siRNA oligonucleotides:  AGGCUUCACCAUCGAGUAA, CCAAGACAACAACGCCAAA, GGAUGAAGUCCUGCGAAC, and UGGUCAUCGUGCAUGGUAA) using the Dharmafect Duo transfection reagent according to the manufacturer's instructions (Thermo Scientific). The ON-TARGETplus nontargeting pool containing four different nontargeting siRNA oligonucleotides (Thermo Scientific) was used as a negative control. At 36 hours after siRNA transfection, the cells were pre-treated with IL-4 for 30 min and stimulated with IFN*γ* and/or LPS for 8 hours before preparation of total RNA for real-time RT-PCR or total cellular lysate for western blotting.

### 2.7. Construction of the Luciferase Reporter Gene

The promoter/enhancer region of the mouse *Nos2* gene (−996~+104; see [23]) was isolated from mouse genomic DNA (Promega, Madison, WI, USA) by PCR using Pfx Ultima DNA polymerase (Invitrogen). The PCR primers are listed in supplemental Table 2. The gene-specific forward and reverse primers contained restriction enzyme sites (*MluI* and* BglII*, resp.); the PCR product was digested with *MluI* and *BglII*, analyzed on a 1.0% agarose gel, and purified using a DNA extraction kit (Qiagen, Valencia, CA, USA) according to the manufacturer's instructions. The purified PCR product (−996~+104) was then subcloned into an *MluI*- and *BglII*-digested pGL2 luciferase reporter plasmid (Promega), and the resulting plasmid was designated as pNOS-996. A series of deletion mutants of the 5′-flanking region of the *Nos2* gene was constructed by restriction enzyme digestion of pNOS-996 (*SmaI* for −772, *SacI* for −333, and *PstI* for −44). Another series of proximal 5′-flanking region of deletion mutants, corresponding to the region between +104 and −143, −86, −62, or −17, was generated by PCR using forward and reverse primers containing *MluI* and* BglII* sites, respectively (supplemental Table 2). The PCR products were digested with *MluI* and* BglII* and subcloned into the pGL2 luciferase reporter plasmid.

Site-specific mutations of the proximal *κ*B site and the OCT site were created by a modification of the two-round PCR method [42]. In brief, the *κ*B site (5′-GGGACTCTCC-3′) was mutated to 5′-ccAC*atcgat*-3, where the lowercase letters indicate the mutant sequences and the italics indicate the *ClaI* site, using two sets of PCR primers containing the mutant sequences (supplemental Table 2). The OCT site (5′-ATGCAAAA-3′) was mutated to 5′-*cgtacg*AA-3′, where the lowercase letters and italics indicate the mutant sequence and *BsiWI* site, respectively, using two sets of primers. The detailed methods for the construction of the mutant reporter constructs are described in supplemental Figure 1. The resulting mutant constructs were confirmed by sequencing.

### 2.8. Transient Transfection and Luciferase Reporter Assay

RAW264.7 cells were seeded into 24-well plates at a density of 1 × 10^4^ cells/well in DMEM supplemented with 10% FBS and cultured for 16 hours prior to transfection. The cells were then transiently transfected with the luciferase reporter plasmids and the pRL-TK reference *Renilla* luciferase plasmid (Promega) using FuGene transfection reagents (Roche) according to the manufacturer's instructions. In some experiments, the CREB-binding protein (CBP) expression vector [43] (kindly proved by Dr. Christopher K. Glass, University of California San Diego) or a control vector (pCMV) was cotransfected with the luciferase reporter plasmid. After 24 hours, the cells were pre-treated with IL-4 for 30 min and stimulated with IFN*γ* and/or LPS for 8 hours. The firefly and *Renilla* luciferase activities were assayed using reagents provided by Promega according to their instructions. For standardization of the transfection efficiency, the luciferase activity from *Nos2* was normalized to the *Renilla* luciferase activity.

### 2.9. Preparation of Cellular Extracts

Nuclear and cytosolic extracts were prepared using a modification of the method of Dignam et al. [44], as described previously [45]. After stimulation, the cells were washed with ice-cold PBS, harvested, and resuspended in 300 *μ*L of hypotonic buffer A (10 mM HEPES, pH 7.9, 10 mM KCl, 0.1 mM EDTA, 0.1 mM EGTA, 1 mM DTT, 1 mM PMSF, and 10 *μ*g/mL of leupeptin, antipain, aprotinin, and pepstatin) for 10 min on ice. The cells were lysed in 0.6% NP-40 by vortexing for 10 seconds. The nuclei were separated from the cytosol by centrifugation at 12,000 ×g for 30 seconds, and the supernatant was saved as a cytosolic fraction. The residual nuclei were washed with 600 *μ*L of buffer A, resuspended in buffer C (20 mM HEPES, pH 7.9, 25% glycerol, 0.4 M NaCl, 1 mM EDTA, 1 mM EGTA, 1 mM DTT, 1 mM PMSF, and 10 *μ*g/mL of leupeptin, antipain, aprotinin, and pepstatin), and briefly sonicated on ice. Nuclear extracts were obtained by centrifugation at 12,000 ×g for 10 min; the protein concentration was measured using the Bradford method [39] with a protein dye reagent (Bio-Rad, Hercules, CA, USA).

### 2.10. Electrophoretic Mobility Shift Assay (EMSA)

The following oligonucleotides (sense strand) were used in the EMSA: wt NOS OCT [33]; 5′-tcgaCAGTTATGCAAAATAGCT-3′; mut NOS OCT, 5′-tacgaCAGTT*CGTA* CGAATAGCT-3′; and wt Ig*κ*B OCT [46], 5′-tcgaTAATAATTTGCATACCT-3′. The underlined sequence and italics represent the consensus sequence for OCT and the mutant sequences, respectively. For the binding reactions, nuclear extracts (5 *μ*g protein) were incubated in 12.5 *μ*L (total volume) containing 20 mM HEPES (pH 7.9), 50 mM KCl, 0.1 mM EDTA, 1 mM DTT, 5% glycerol, 200 *μ*g/mL BSA, and 1.25 *μ*g of poly(dI-dC) for 15 minutes at room temperature. [^32^P]-Labeled oligonucleotide (0.5 ng, 5 × 10^5^ cpm) was then added to the reaction mixture and incubated for 15 minutes at room temperature. The reaction products were analyzed by electrophoresis through a 5% polyacrylamide gel with 0.25 × TBE buffer (22.3 mM Tris, 22.2 mM borate and 0.5 mM EDTA). In some experiments, rabbit antibodies against Oct-1, Oct-2, and Bob-1/BOB.1 were added prior to electrophoresis. The dried gels were analyzed by autoradiography and phosphorescence detection.

### 2.11. Western Blotting Analysis

Cells were harvested after stimulation and resuspended in RIPA buffer [0.1% SDS, 1% NP-40, 5 mM EDTA, 0.5% sodium deoxycholate, 150 mM NaCl, 50 mM HEPES (pH 8.0), 2 *μ*g/mL leupeptin, 20 mg/mL aprotinin, 20 *μ*g/mL Na_3_VO_4_, 10 mM NaF, 1 mM PMSF, and 2 mM DTT] and centrifuged at 4°C, 12,000 ×g for 10 min; the supernatant was recovered as the total cell lysate. In some experiments, nuclear extracts were used as the samples. The protein concentration was measured by the Bradford method [39] using a protein dye reagent (Bio-Rad). Equal amounts of protein were denatured in SDS sample buffer [62.5 mM Tris-HCl (pH 6.8) containing 2% SDS, 20% glycerol, 5% 2-mercaptoethanol, and 0.2% bromophenol blue], separated by SDS-polyacrylamide gel electrophoresis, and transferred to polyvinylidene fluoride (PVDF) membranes (Millipore, Bedford, MA, USA). The membranes were blocked with 5% non-fat milk in TBS-T [50 mM Tris-HCl (pH 7.4) containing 150 mM NaCl and 0.1% Tween-20], incubated overnight with primary antibodies at 4°C, and washed three times with TBS-T. The blots were then incubated for 1 hour at room temperature with secondary antibodies conjugated to horseradish peroxidase and washed again with TBS-T. The blots were developed using a SuperSignal West Pico chemiluminescence substrate kit (Pierce, Rockford, IL, USA).

### 2.12. Statistical Analysis

Student's *t* tests for paired data were used to test for statistically significant differences with Prism 5 software (GraphPad Software, La Jolla, CA, USA). The results are expressed as the mean ± SEM of at least three experiments. A *P* value less than 0.05 was considered to be statistically significant.

## 3. Results

### 3.1. Inhibition of IFN*γ*- and/or LPS-Induced *Nos2* mRNA Expression by IL-4 Depends on Stat6 in RAW264.7 Cells

**Figure 1:**
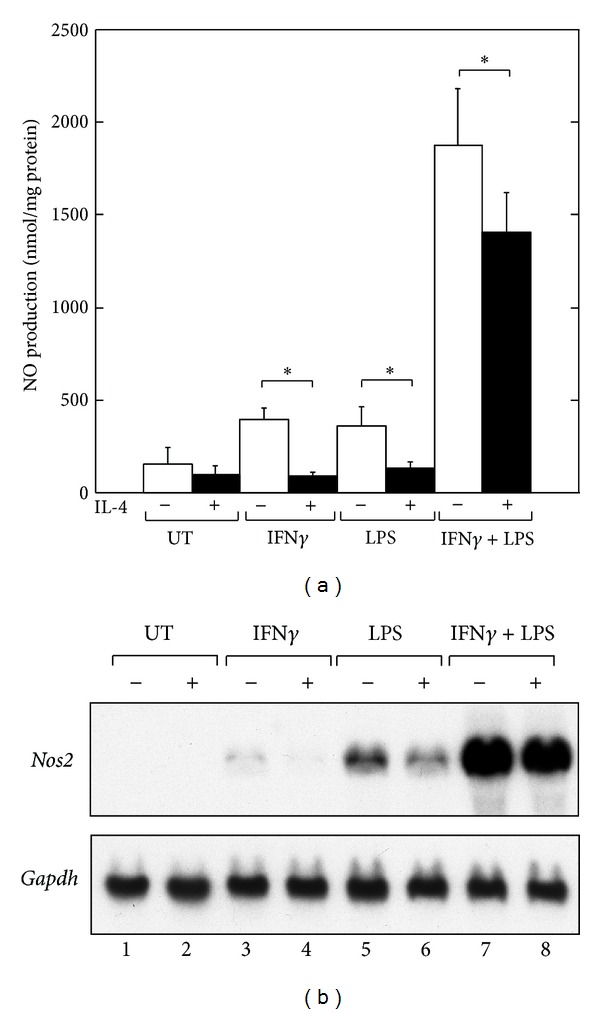
IL-4 inhibits IFN*γ*- and/or LPS-induced NO production and *Nos2* mRNA expression in RAW264.7 cells. (a) RAW264.7 cells were treated with medium alone (untreated, UT) or IL-4 (10 ng/mL) for 30 min prior to stimulation with IFN*γ* (10 ng/mL) and/or LPS (100 ng/mL). The culture supernatants were harvested and assessed for NO production by the Griess assay. The protein concentrations of the residual cells in the cultures were also determined. Each column and bar represents the mean ± SEM of three independent experiments. The asterisks denote a statistically significant difference compared to the cultures with IL-4, (*P < 0.05; Student's *t* test). (b) RAW264.7 cells were treated with medium alone or IL-4 (10 ng/mL) for 30 min prior to stimulation with IFN*γ* (10 ng/mL) and/or LPS (100 ng/mL) for 8 hours before the preparation of total RNA and analysis of the *Nos2* mRNA level by northern hybridization. The data shown are representative of three independent experiments.

We initially assessed whether IL-4 inhibits the NO production induced by IFN*γ* and/or LPS in the macrophage-like cell line RAW264.7 (Figure 1(a)). RAW264.7 cells were treated with medium alone or IL-4 for 30 min prior to stimulation with IFN*γ* and/or LPS for 48 hours, and the culture supernatants were then harvested for the analysis of NO production. As shown in Figure 1(a), IL-4 significantly inhibited NO production in the RAW264.7 cells stimulated with IFN*γ* and/or LPS. To examine the inhibitory effect of IL-4 on IFN*γ* and/or LPS-induced *Nos2* mRNA expression, the cells were pretreated with IL-4 for 30 min and stimulated with IFN*γ* and/or LPS for 8 hours; total RNA was then prepared and analyzed by northern hybridization (Figure 1). In agreement with previous studies [12, 28, 34–36], pretreatment with IL-4 inhibited IFN*γ* and/or LPS-induced *Nos2* mRNA expression.

**Figure 2:**
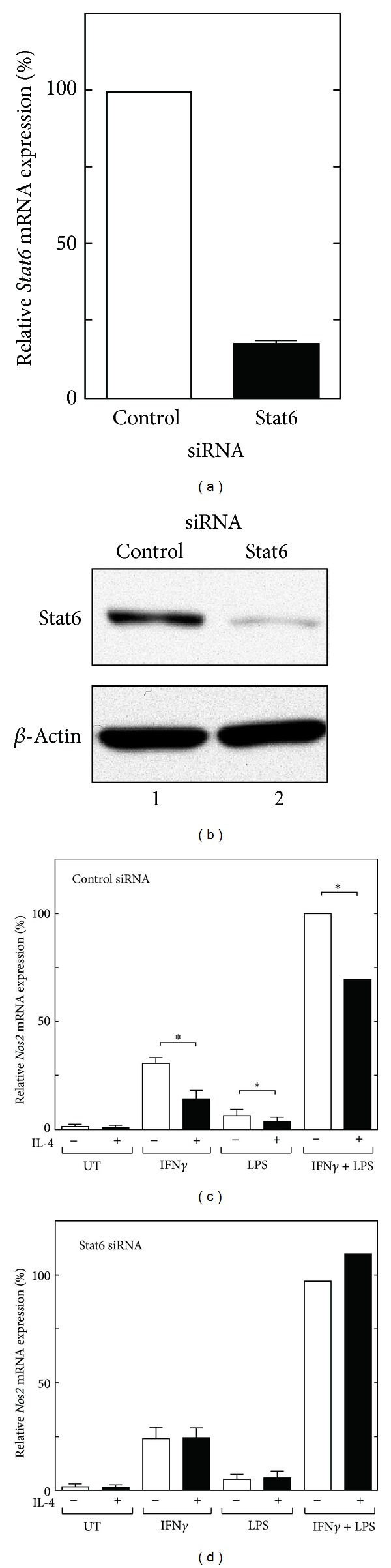
Stat6 is required for the IL-4-mediated inhibition of *Nos2* gene expression in RAW264.7 cells. (a) RAW264.7 cells were transfected with control siRNA (100 nM) or Stat6 siRNA (100 nM) for 36 hours; total RNA was then prepared for quantitative real-time RT-PCR. Each column and bar represents the mean ± SEM of three independent experiments. (b) RAW264.7 cells were transfected with siRNA, as described above, and then used to prepare total cellular lysates for a western blot analysis using an anti-Stat6 antibody. (c, d) RAW264.7 cells were transfected with control siRNA (100 nM) or Stat6 siRNA (100 nM). Thirty-six hours after transfection, the cells were either left untreated (UT) or treated with IL-4 (10 ng/mL) for 30 min prior to stimulation with IFN*γ* (10 ng/mL) and/or LPS (100 ng/mL) for 8 hours before the preparation of total RNA for quantitative real-time RT-PCR. The relative *Nos2* mRNA expression levels are shown as percentages of the activity of cells transfected with the control siRNA and stimulated with IFN*γ* and LPS. Each column and bar represents the mean ± SEM of three independent experiments. The asterisks denote a statistically significant difference compared to the cultures treated with IL-4 (*P* < 0.05, Student's *t* test).

The biological activity of IL-4 has been shown to be largely mediated by the transcription factor Stat6 [16, 17]. Thus, to determine whether the inhibition of *Nos2* gene expression by IL-4 is mediated by Stat6, we knocked down Stat6 using a SMARTpool of siRNA targeting mouse Stat6. Real-time RT-PCR and western blotting analyses confirmed that siRNA-mediated Stat6 knockdown significantly decreased the levels of Stat6 transcription and protein expression compared to the control siRNA treatment (Figures 2(a) and 2(b)). In agreement with the decreased Stat6 level, the IL-4-mediated inhibition of *Nos2* gene expression induced by IFN*γ* and/or LPS was abolished in the cells transfected with Stat6 siRNA (Figure 2(d)). These results indicate that the IL-4-mediated inhibition of *Nos2* gene expression depends on Stat6.

### 3.2. Analysis of the *Nos2* Regulatory Region for IL-4-Mediated Inhibition

**Figure 3:**
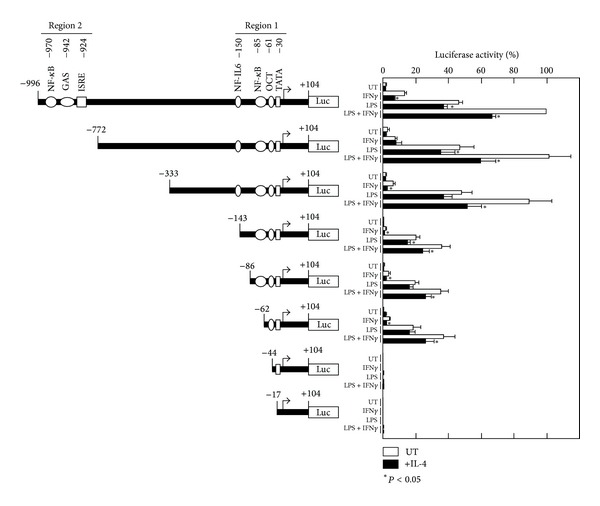
Deletion analysis of the mouse *Nos2* enhancer/promoter region by luciferase reporter assays in RAW264.7 cells. The diagram on the left shows the wild-type (pNOS-996) and deletion constructs of the *Nos2* luciferase reporter. The numbers above the enhancer/promoter region refer to the nucleotide positions relative to the transcriptional start site of the mouse *Nos2* gene. NF-*κ*B, nuclear factor kappa B; GAS, gamma-IFN activation sequence; ISRE, interferon-stimulated responsive element; NF-IL-6, nuclear factor IL-6; OCT, octamer transcription factor; TATA, TATA-box. RAW264.7 cells were transiently transfected with wild-type or mutant *Nos2* luciferase reporter constructs. Twenty-four hours after transfection, the cells were either left untreated (UT) or treated with IL-4 (10 ng/mL) for 30 min prior to stimulation with IFN*γ* (10 ng/mL) and/or LPS (100 ng/mL) for 8 hours before measurement of the luciferase activity. The relative luciferase activities are shown as percentages of the activity in cells transfected with the wild-type construct (pNOS-996) and stimulated with IFN*γ* and LPS. Each column and bar represents the mean ± SEM of five independent experiments. The asterisks denote a statistically significant difference compared to the cultures with IL-4 (*P* < 0.05, Student's *t* test).

To examine the mechanism by which IL-4-induced Stat6 inhibits *Nos2* gene expression, we isolated the 5′-regulatory region of the mouse *Nos2* gene (−996~+104 from the transcriptional start site) from mouse genomic DNA using PCR. We then cloned the amplified fragment into the pGL2 luciferase reporter plasmid (designated as pNOS-996) and analyzed the luciferase activity in a transient transfection assay in RAW264.7 cells (Figure 3). When the cells were stimulated with IFN*γ* alone, a small but significant increase in luciferase activity was observed, whereas pre-treatment with IL-4 inhibited this IFN*γ*-induced luciferase activity by 50%. Stimulation with LPS alone strongly induced luciferase activity; IL-4 also inhibited the LPS-induced luciferase activity, though the magnitude of the inhibition was smaller than that of the IFN*γ*-induced luciferase activity. A synergistic induction of luciferase activity was observed when the cells were stimulated with a combination with IFN*γ* and LPS, and pre-treatment with IL-4 also inhibited this luciferase activity.

To identify the regulatory region responsible for the IL-4-mediated inhibition of *Nos2*, a series of 5′ deletion mutants of the *Nos2* gene was analyzed. The deletion of the distal enhancer elements (region 2) diminished the IFN*γ*-induced luciferase activity (pNOS-772, pNOS-333); however, the LPS- and IFN*γ* plus LPS-stimulated luciferase activities were comparable to the activity of the full-length promoter construct. Furthermore, pre-treatment with IL-4 inhibited the luciferase activity induced by IFN*γ* and LPS in cells transfected with these reporter constructs. Deletion of the NF-IL-6 site (pNOS-143) diminished the constitutive activity and the IFN*γ* and/or LPS-induced luciferase activity, indicating that this site contributes to the overall promoter activity; the IL-4-mediated inhibition of luciferase activity was still observed with this construct. Although the deletion of the NF-*κ*B site (pNOS-62) only marginally affected the luciferase activity, deletion of the octamer (OCT) site almost completely abolished the activity (pNOS-44).

### 3.3. The Octamer Transcription Factor Binding Site Is Involved in the IL-4-Mediated Inhibition of *Nos2* Gene Expression

**Figure 4:**
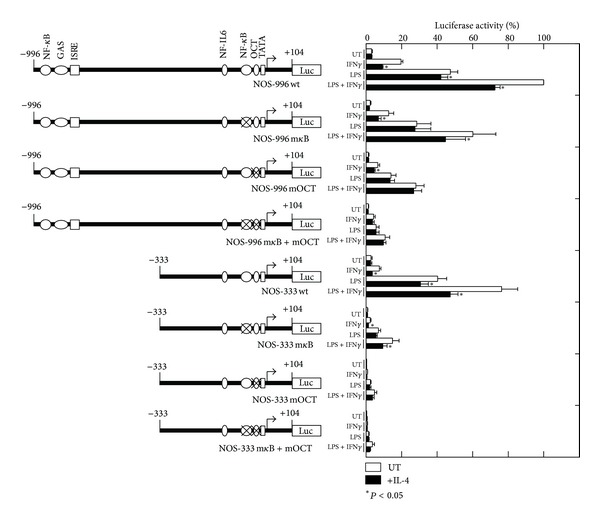
Mutational analysis of the mouse *Nos2* enhancer/promoter region by luciferase reporter assays in RAW264.7 cells. The diagram on the left shows the wild-type (pNOS-996 and pNOS-333) and mutant *Nos2* luciferase reporter constructs. Mutations of the NF-*κ*B and/or OCT sites in the constructs are also indicated. RAW264.7 cells were transiently transfected with the wild-type or the mutant *Nos2* luciferase reporter construct, as described above. The relative luciferase activities are shown as percentages of the activity in cells transfected with the wild-type construct (pNOS-996) and stimulated with IFN*γ* and LPS. Each column and bar represents the mean ± SEM of three independent experiments. The asterisks denote a statistically significant difference compared to the cultures treated with IL-4 (*P* < 0.05, Student's *t* test).

Because the OCT site in the proximal promoter region appears to be critical for the transcriptional regulation of the *Nos2* gene, site-specific mutations of this site, and the *κ*B sites were created in the full-length pNOS-996 luciferase construct and analyzed for their luciferase activity (Figure 4). Although mutation of the proximal *κ*B site diminished the LPS-induced luciferase activity and IL-4-mediated inhibition of luciferase activity, an inhibitory effect of IL-4 on the IFN*γ*- or IFN*γ* plus LPS-induced luciferase activity was still observed. Nonetheless, mutation of the OCT site further diminished the IFN*γ*- and/or LPS-induced luciferase activity, and the inhibitory effect of IL-4 was also diminished, suggesting that the OCT site is responsible for mediating the inhibitory effect of IL-4. To determine the importance of the OCT site in the positive and negative regulation of the *Nos2* gene, the *κ*B and OCT sites were mutated within the context of pNOS-333 in which the distal enhancer region had been deleted. The mutation of *κ*B markedly reduced the LPS-induced luciferase activity, though the inhibitory effect of IL-4 was still observed in the cells stimulated with IFN*γ*- or IFN*γ* plus LPS. When the OCT site was mutated, both the luciferase activity induced by IFN*γ* and/or LPS and the inhibitory effect of IL-4 were markedly reduced. These results indicate that the OCT site appears to mediate the inhibitory effect of IL-4 in the transcriptional regulation of the *Nos2* gene in RAW276.7 cells.

**Figure 5:**
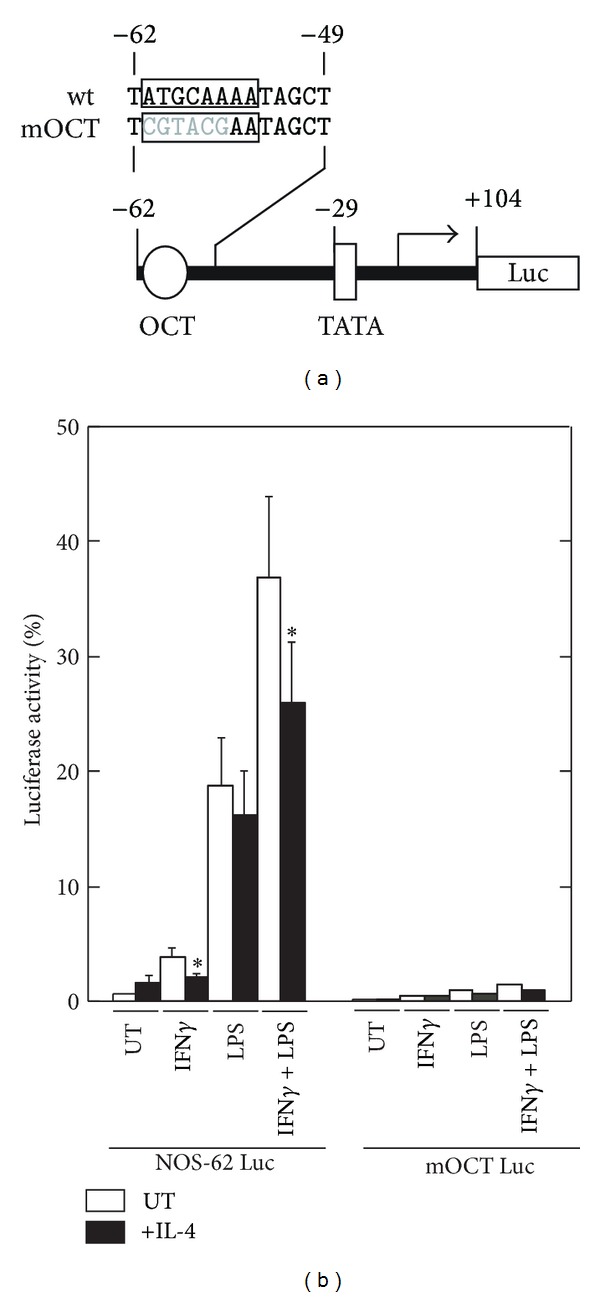
The OCT site in the *Nos2* promoter region is required for IFN*γ*- and LPS-induced promoter activity. (a) The diagram shows the wild-type (wt) and mutant sequences of the OCT site (mOCT) in the *Nos2* minimum promoter region. The numbers above the promoter region refer to the nucleotide positions relative to the transcription start site of the mouse *Nos2* gene. (b) RAW264.7 cells were transiently transfected with the NOS-62 luciferase reporter construct or a mutant construct containing a mutated OCT site (mOCT), as described above. The relative luciferase activities are shown as percentages of the activity in cells transfected with the wild-type construct (pNOS-996) and stimulated with IFN*γ* and LPS. Each column and bar represents the mean ± SEM of three independent experiments. The asterisks denote a statistically significant difference compared to the cultures treated with IL-4 (*P* < 0.05, Student's *t* test).

To further confirm the importance of the OCT site in the regulation of *Nos2*, this site was mutated by site-directed mutagenesis within the context of the minimum promoter construct (pNOS-62), which contains the OCT site, a TATA box, and 104 bp of the 5′-untranslated region; the ability of this construct to mediate the response to IFN*γ* and/or LPS was then assessed in transient transfection assays (Figure 5). Mutation of the OCT site almost completely abolished the luciferase activity induced by IFN*γ* and/or LPS, indicating that the OCT site is required for the transcriptional activation of the minimal promoter construct and that the transcriptional repression by IL-4 is mediated through this site.

### 3.4. Analysis of OCT Binding Activity in Nuclear Extracts Prepared from RAW264.7 Cells

**Figure 6:**
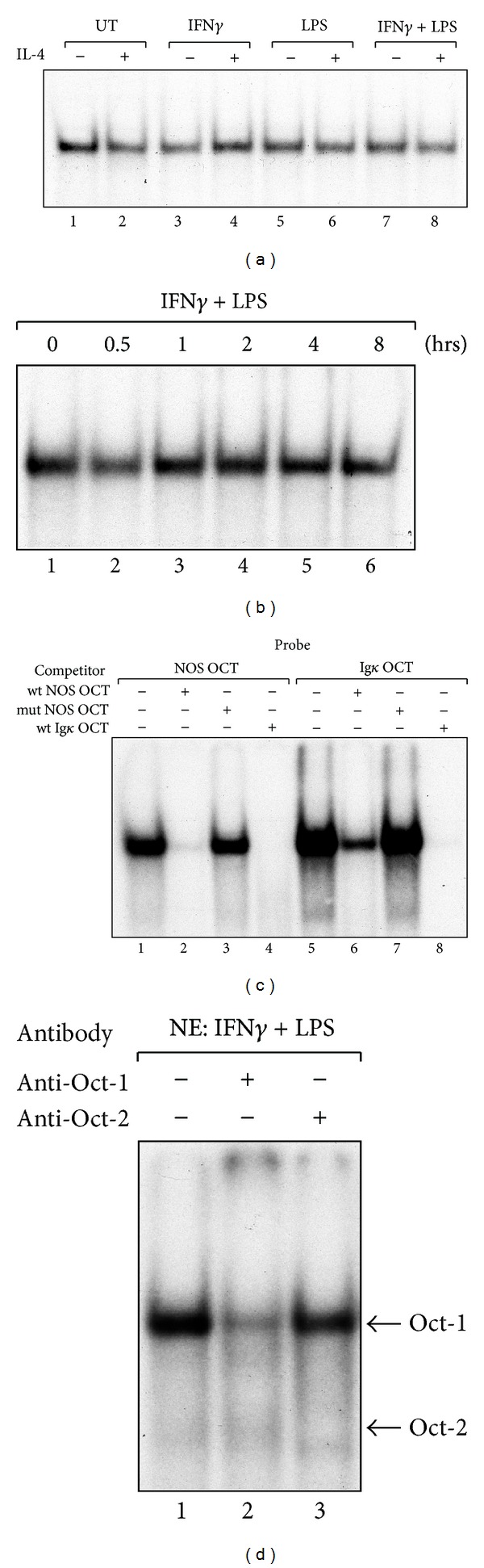
Analysis of OCT DNA-binding activity in nuclear extracts from RAW264.7 cells. (a) Effect of IL-4 treatment on OCT DNA-binding activity. RAW264.7 cells were treated with medium alone or IL-4 (10 ng/mL) for 30 min prior to stimulation with IFN*γ* (10 ng/mL) and/or LPS (100 ng/mL) for 4 hours before the preparation of nuclear extracts. The OCT binding activity was assessed by EMSA. (b) OCT DNA-binding activity in nuclear extracts from RAW264.7 cells treated with IFN*γ* and LPS. RAW264.7 cells were cultured in the presence of IFN*γ* (10 ng/mL) and LPS (100 ng/mL) for the indicated time prior to the preparation of nuclear extracts. In total, 10 *μ*g of each nuclear extract was analyzed for OCT binding activity by EMSA. (c) Analysis of OCT DNA-binding affinity to *Nos2* OCT by an oligonucleotide competition assay. Nuclear extracts were prepared from RAW264.7 cells stimulated with IFN*γ* (10 ng/mL) and LPS (100 ng/mL) for 30 min. The OCT DNA-binding activity was determined by EMSA using radio-labeled OCT oligonucleotides corresponding to the *Nos2* OCT (NOS OCT) site or the immunoglobulin *κ* chain OCT site (Igk OCT) in the presence or absence of a 25-fold excess of unlabeled wild-type (wt) or mutant (mut) oligonucleotide, as indicated. (d) Antibody super-shift assay for *Nos2* OCT. Nuclear extracts (NE) from RAW264.7 cells stimulated with IFN*γ* (10 ng/mL) and LPS (100 ng/mL) for 30 min were incubated with the indicated antibodies (1 *μ*g each) before analysis of the binding activity, as described above.

To examine whether IL-4 induces or modulates the nuclear factor(s) that specifically bind(s) to the OCT sequence, nuclear extracts were prepared from RAW264.7 cells and analyzed by EMSA using radiolabeled oligonucleotides corresponding to the OCT sequence (Figure 6). Constitutive DNA binding activity was observed in nuclear extracts from untreated cells, and this binding activity was not enhanced by either IFN*γ* or LPS alone or in combination (Figure 6(a) lanes 1, 3, 5, and 7). In addition, these constitutive OCT-binding activities were not modified by IL-4 treatment (lanes 2, 4, 6, and 8). A time-course experiment showed that no significant increase in the OCT-binding activity was observed in the nuclear extracts from RAW264.7 cells treated with IFN*γ* and LPS (Figure 6(b)). The observed OCT-binding activity was specifically competed with a wild-type *Nos2* OCT (wt NOS OCT) oligonucleotide (Figure 6(c), lane 2) and a consensus OCT motif from the immunoglobulin kappa chain (*Ig*κ**) gene (wt Ig*κ* OCT) [46] (lane 4), whereas a mutant *Nos2* OCT oligonucleotide (lane 3) did not compete in our EMSA. Although the OCT-binding activity was competed with the wild-type *Nos2* OCT when the consensus* Ig*κ** OCT motif was used as the probe (lane 6), the competition was less efficient compared to the *Ig*κ**OCT motif (lane 8).

An antibody super-shift assay was then performed to identify the transcription factor that binds to the *Nos2* OCT site (Figure 6(d)). An antibody against octamer transcription factor-1 (Oct-1) super-shifted the OCT-binding activity (lane 2), and an antibody against Oct-2 diminished the band(s) migrating below the Oct-1 complex (lane 3). These results indicate that the OCT-binding complex mainly contains constitutively expressed Oct-1 and some Oct-2 and that treatment with IL-4 had no effect on the binding of Oct proteins to the *Nos2* OCT site. We also confirmed that IL-4 had no inhibitory effect on Oct-1 or Oct-2 mRNA and protein expression in RAW 264.7 cells (supplemental Figure 2).

### 3.5. Overexpression of CBP Partially Attenuates the IL-4-Mediated Inhibition of *Nos2* Promoter Activity

**Figure 7:**
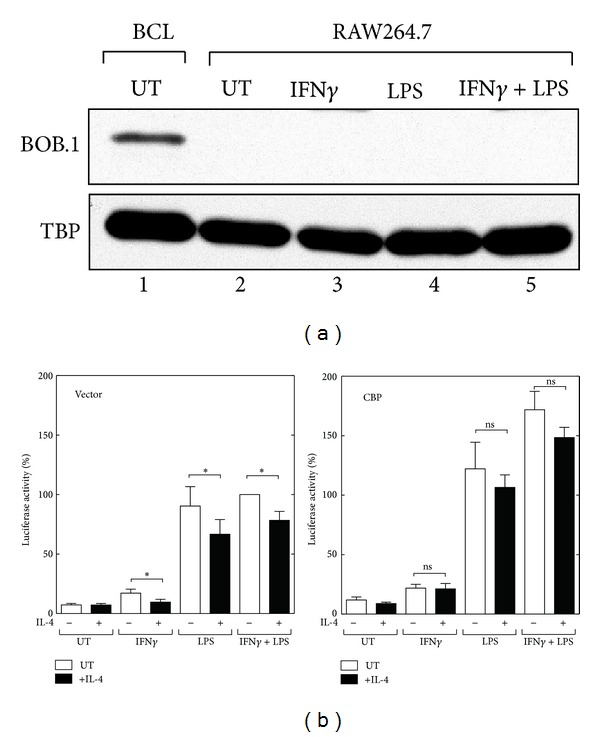
Overexpression of the coactivator CBP partially attenuates the IL-4-mediated inhibition of *Nos2* promoter activity in RAW264.7 cells. (a) Analysis of endogenous BOB.1 expression in RAW264.7 cells. The cells were treated with medium alone (UT) or IFN*γ* (10 ng/mL) and/or LPS (100 ng/mL) for 8 hours before the preparation of nuclear extracts. Twenty micrograms of nuclear extract was analyzed by western blotting using an antibody against BOB.1 or an antibody against TATA-binding protein (TBP), which was used as a loading control. Nuclear extracts from the mouse leukemia cell line BCL1-B20 (BCL) were used as a positive control for BOB.1 expression (lane 1). (b) RAW264.7 cells were transiently co-transfected with either the empty vector or wild-type CBP expression plasmid and the pNOS-62 luciferase reporter construct. Twenty-four hours after transfection, the cells were treated with medium alone (untreated: UT) or IL-4 (10 ng/mL) for 30 min prior to stimulation with IFN*γ* (10 ng/mL) and/or LPS (100 ng/mL) for 8 hours before the measurement of luciferase activity. The relative luciferase activities are shown as the percentage of the activity of cells transfected with the empty vector and stimulated with IFN*γ* and LPS. Each column and bar represents the mean ± SEM of three independent experiments. The asterisks denote a statistically significant difference compared to the cultures treated with IL-4 (*P* < 0.05, Student's *t* test; ns, not significant).

Oct-1 and Oct-2 have been shown to interact with the coactivator BOB.1/OBF.1 (official symbol: Pou2af1) and to promote OCT-dependent transcription [47–49]. Because the *Nos2* OCT binding activity was not modified by IL-4, a coactivator and/or cofactor that interacts with Oct-1 might be the target of IL-4-mediated inhibition. To examine the levels of endogenous BOB.1 protein in RAW264.7 cells, western blots were performed with lysates from cells stimulated with IFN*γ* and/or LPS (Figure 7(a)). Although constitutive expression of the BOB.1 protein was observed in a mouse B cell line BCL1-B20 (lane 1), no detectable levels of BOB.1 protein were observed in the IFN*γ* and/or LPS-stimulated RAW264.7 cells (lanes 2~5). These results suggest that a coactivator/cofactor other than BOB.1 is involved in the Oct-1-dependent transcriptional activation and IL-4-mediated inhibition of the *Nos2 *gene.

Because IL-4-induced Stat6 interacts with coactivator CREB-binding protein (CBP) in macrophages and CBP is expressed in mouse macrophages [19], we transfected a CBP expression vector into RAW264.7 cells and investigated the inhibitory effect of IL-4 on *Nos2* promoter activity. As shown in Figure 7(b), transfection with the CBP expression vector potentiated the IFN*γ*- and LPS-induced *Nos2* promoter activity and partially attenuated the IL-4-mediated inhibition of promoter activity induced by IFN*γ* and/or LPS. Taken together, these results suggest a model in which the IL-4-mediated inhibition of *Nos2* promoter activity depends on a coactivator/cofactor that functionally promotes the Oct-1-dependent transcriptional activation of the *Nos2* gene; furthermore, the transcriptional repression by IL-4 might be mediated by the sequestration of this coactivator/cofactor by IL-4-induced Stat6.

## 4. Discussion

We and others have previously shown that Stat6 functions as a negative regulator of the anti-inflammatory activity of IL-4 or IL-13 in INF*γ*-induced macrophage gene expression [19–21, 50]. Although Stat6 is required for the IL-4 or IL-13-mediated inhibition of mouse *Nos2* induced by IFN*γ* and LPS in mouse macrophages [50], the molecular mechanisms involved in the Stat6-mediated inhibition remain to be fully elucidated. In the present study, we explored the mechanism involved in the IL-4-mediated inhibition of the *Nos2* gene in the mouse macrophage-like cell line RAW264.7. We initially confirmed that Stat6 is necessary for the IL-4-mediated inhibition of *Nos2* gene expression using siRNA knockdown (Figure 2). We then investigated the mechanism by which IL-4-induced Stat6 inhibits the transcriptional activation of the *Nos2* gene using a transient transfection assay with a luciferase reporter gene containing a series of 5′-deletion mutants and site-directed mutants of the* Nos2* regulatory region. Our results indicate that an OCT site located in the proximal promoter region of the *Nos2* gene is required for both the transcriptional activation induced by IFN*γ*/LPS and transcriptional repression by IL-4 in RAW264.7 cells. These conclusions are based on the following observations. Mutation of the OCT site within the context of the full-length *Nos2* promoter construct (pNOS-996 Luc) markedly reduced the promoter activity induced by INF*γ* and/or LPS and the IL-4-mediated inhibition of promoter activity (Figure 4). A minimal promoter construct of the mouse *Nos2* gene (pNOS-62 Luc), which contains an OCT site and TATA box, retained the responsiveness to both IFN*γ*/LPS and IL-4, whereas mutation of the OCT site almost completely abolished this responsiveness (Figure 5).

The OCT site in the *Nos2* proximal promoter region has been shown to play a critical role in LPS-induced *Nos2* gene expression [31–33, 51–53]. The octamer motif (ATGCAAAT) is a conserved DNA binding element found in the promoter/enhancer region of many genes, including such ubiquitously expressed genes as the histone H2B gene, small nuclear RNA genes, and tissue-specific genes [54]. Members of the POU homeodomain family Oct-1 and Oct-2 recognize the OCT site of the mouse *Nos2* gene [32, 33, 51–53]. The results presented in this study are consistent with the previous observation that the octamer motif in the *Nos2* promoter is required for LPS-induced promoter activity. Furthermore, our findings demonstrate the essential role of this motif in IFN*γ*-induced promoter activity and the IL-4-mediated negative regulation of promoter activity. To our knowledge, this is the first demonstration that the OCT site in the *Nos2 *promoter region mediates transcriptional repression by IL-4 in mouse macrophages.

Previous research by Lu showed that LPS induces Oct-2 expression in RAW264.7 cells and that trichostatin A, a histone deacetylase inhibitor, suppresses LPS-induced *Nos2* expression by inhibiting LPS-induced Oct-2 expression [53], suggesting that Oct-2 activation is a crucial step for the transcriptional activation of the *Nos2* gene. Although we also observed an increase in Oct-2 mRNA and protein expression in RAW264.7 cells treated with LPS (supplemental Figure 2), IL-4 inhibition of the *Nos2* gene was not due to the down-regulation of Oct-1 or Oct-2 expression: EMSA showed that, although octamer binding activities, which are mainly due to Oct-1 and a small amount of Oct-2, were observed in RAW264.7 cell nuclear extracts, IL-4 had no effect on the observed octamer biding activity (Figure 6). Furthermore, real-time RT-PCR and western blotting analyses showed that IL-4 had no inhibitory effect on the expression of the Oct-1 and Oct-2 mRNAs and proteins (supplemental data). These lines of evidence indicate that the down-regulation of Oct-1 and Oct-2 expression is not likely to be the mechanism by which IL-4 inhibits *Nos2* gene transcriptional activity.

The transcriptional activities of Oct-1 and Oct-2 are known to be enhanced by the lymphocyte-specific coactivator BOB.1/OBF.1 [47–49, 55]. Therefore, it is conceivable that a coactivator or cofactor that functionally interacts with Oct-1/Oct-2 might be a target for the IL-4-mediated transcriptional repression of *Nos2*, that is, IL-4-activated Stat6 competes with a coactivator/cofactor that interacts with Oct-1. Thus, if the inhibition is mediated by competition for this coactivator/cofactor, overexpression of the coactivator/cofactor would attenuate the transcriptional repression by IL-4. However, BOB.1 has been shown to be a lymphocyte-specific coactivator, and macrophage-lineage cells do not express BOB.1 [48, 56]; we also confirmed that RAW264.7 cells did not express BOB.1, even upon stimulation with IFN*γ* and/or LPS (Figure 7(a)). These findings suggest that a coactivator/cofactor other than BOB.1 that functionally interacts with Oct-1 or Oct-2 may be a molecular target for the IL-4-mediated inhibition of the *Nos2* gene. We have previously shown that IL-4-induced Stat6 interacts with the coactivator CBP in macrophages; indeed, CBP is actually expressed in mouse macrophages [19]. Therefore, we tested the possibility that transfection with a CBP expression vector would affect the IL-4-mediated inhibition of *Nos2* promoter activity. Transfection with a CBP expression vector partially attenuated the inhibitory effect of IL-4 on IFN*γ*/LPS-induced promoter activity, suggesting that CBP and another coactivator/cofactor that functionally interacts with Oct-1 may participate in the transcriptional repression. Oct-1 has been shown to interact with components of the general transcriptional machinery, including TBP, TFIIB, and TFIIH [57–60]. Further studies are required to examine whether IL-4-activated STAT6 directly or indirectly interacts with these components and/or other coactivators/cofactors and inhibits the Oct-1-dependent transcriptional regulation of the *Nos2* gene.

Nitric oxide produced by iNOS is a signature molecule involved in the classical activation of M1 macrophages. iNOS exerts antitumor and microbicidal activities against intracellular pathogens and functions as a potential host-destructive mediator [3, 61]. The functional competence of M1 macrophages, which is generally induced by such TLR ligands as LPS and the T-cell derived cytokine IFN*γ*, is negatively regulated by anti-inflammatory cytokines, including IL-4 and IL-13. Both cytokines, which are known to induce the alternative activation pathway in M2 macrophages, have been shown to regulate NO production by inducing *arginase I*, which catalyzes the hydrolysis of L-arginine, a common substrate of iNOS, and thereby down-regulates NO synthesis by competing with the substrate [8]. Because the induction of *arginase I* by IL-4/IL-13 is also regulated by Stat6 [62], IL-4/IL-13-induced Stat6 appears to actively direct macrophages toward the M2 phenotype by inducing the expression of genes that are involved in the M2 phenotype, such as *arginase I*, and by inhibiting genes involved in the M1 phenotype, such as *Nos2*.

## 5. Conclusions

In conclusion, we demonstrate that the negative regulation of the mouse *Nos2* gene by IL-4 in a mouse macrophage cell line depends on Stat6. Our analysis of the 5′-flanking regulatory region of the *Nos2* gene demonstrated that the OCT site in the proximal promoter region is required for negative regulation by IL-4. Our results suggest a model in which a coactivator/cofactor that functionally interacts with Oct-1 is a molecular target for the IL-4-mediated inhibition of the *Nos2* gene and that IL-4-activated Stat6 represses Oct-1-dependent transcription by competing with this coactivator/cofactor.

## Supplementary Material

Supplemental Figure 1: Schematic outline of the mutagenesis protocol for the construction of the mutant Nos2 luciferase reporter plasmid.
Supplemental Figure 2: Effect of IL-4 on Oct-1 and Oct-2 mRNA and protein expression in RAW264.7 cells stimulated with IFN*γ* and LPS
Supplemental Table 1: Oligonucleotide sequences of primers for real-time PCR analysis.
Supplemental Table 2: Oligonucleotides sequences of PCR primers for construction of the Nos2 luciferase reporter constitutes.
