# Supplementary material for: Anti-Inflammatory Cytokine Interleukin-4 Inhibits Inducible Nitric Oxide Synthase Gene Expression in the Mouse Macrophage Cell Line RAW264.7 through the Repression of Octamer-Dependent Transcription
Source: Mediators Inflamm. 2013 Dec 29;2013:369693. doi: 10.1155/2013/369693 (PMC3891534; doi:10.1155/2013/369693)
Supplement: Supplementary file 1 — Supplemental Figure 1: Schematic outline of the mutagenesis protocol for the construction of the mutant Nos2 luciferase reporter plasmid. Supplemental Figure 2: Effect of IL-4 on Oct-1 and Oct-2 mRNA and protein expression in RAW264.7 cells stimulated with IFNγ and LPS Supplemental Table 1: Oligonucleotide sequences of primers for real-time PCR analysis. Supplemental Table 2: Oligonucleotides sequences of PCR primers for construction of the Nos2 luciferase reporter constitutes. [file 369693.f1.pdf]

Supplemental figures and tables

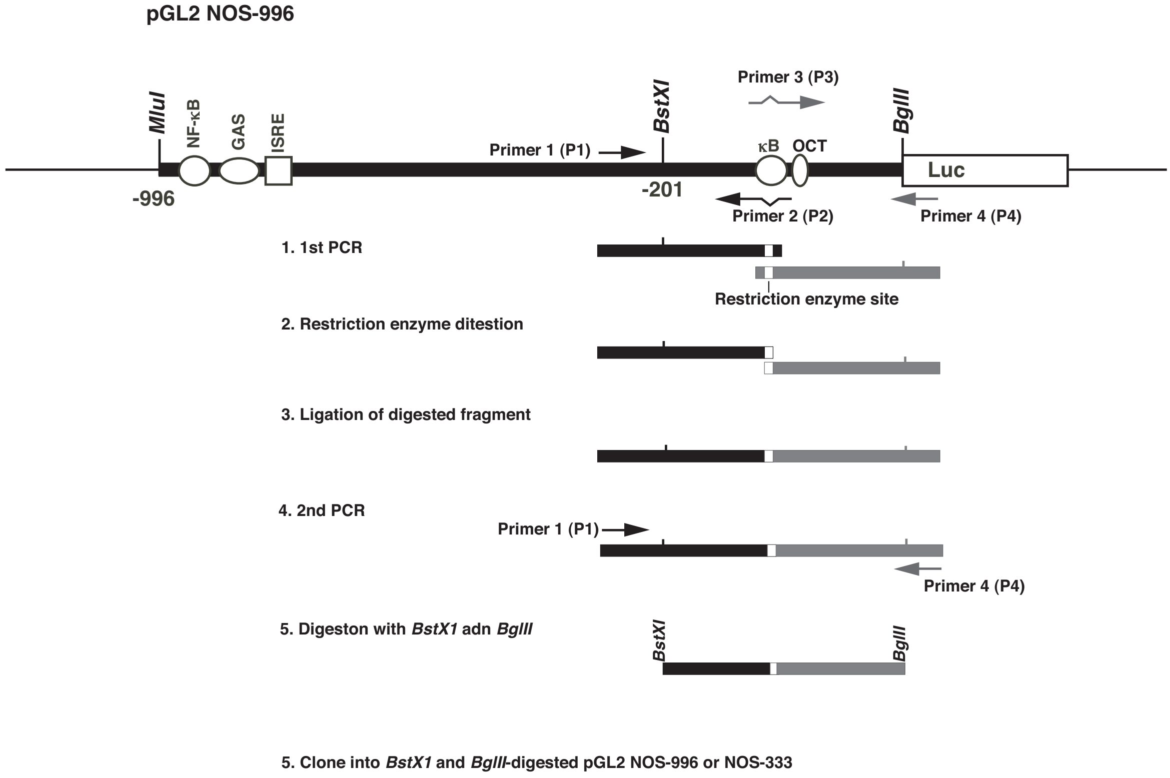

Supplemental Figure 1 Schematic outline of the mutagenesis protocol for the construction of the mutant *Nos2* luciferase reporter plasmid.

The first PCR reactions were performed using two sets of mutagenesis primers containing restriction enzyme sites in the κB site or the OCT site to generate the upstream (between primer 1 and primer 2) and downstream (between primer 3 and primer 4) mutagenesis fragments. The 50-µl reaction mixtures contained 10 pmol of each forward and reverse primer, 0.5 µl Pfx Ultima DNA polymerase (Invitrogen), 50 ng plasmid DNA template (pGL2 NOS-996), and 200 µM of dNTPs in the 1× DNA polymerase buffer supplied by the manufacturer. The first PCR reactions were performed with 25 cycles of amplification using the following reaction conditions: 94 °C for 50 s (except for a 4-min first cycle), 60 °C for 50 s, and 72 °C for 60 s, with a final extension step for 7 min at 72 °C. After completion of the first PCR, each amplified PCR fragment was digested with *ClaI* for the mutant κB site or *BsiWI* for the mutant OCT site, purified by agarose gel electrophoresis, and ligated with T4 ligase. The ligated fragment was used as the template for the second PCR using primer 1 (P1) and primer 4 (P4). The second PCR reaction was performed with the same composition and under the same conditions as the first PCR reaction, except that the extension time was 90 s at 72 °C. The amplified fragments were digested with *BstXI* and *BglII*, purified by agarose gel electrophoresis, and ligated into the parental pNOS-996 or pNOS-333 luciferase reporter plasmid, which had been digested with *BstXI and BglII*. The resulting ligation mixture was used to transform competent cells, and several colonies were selected for the analysis of the nucleotide sequences.

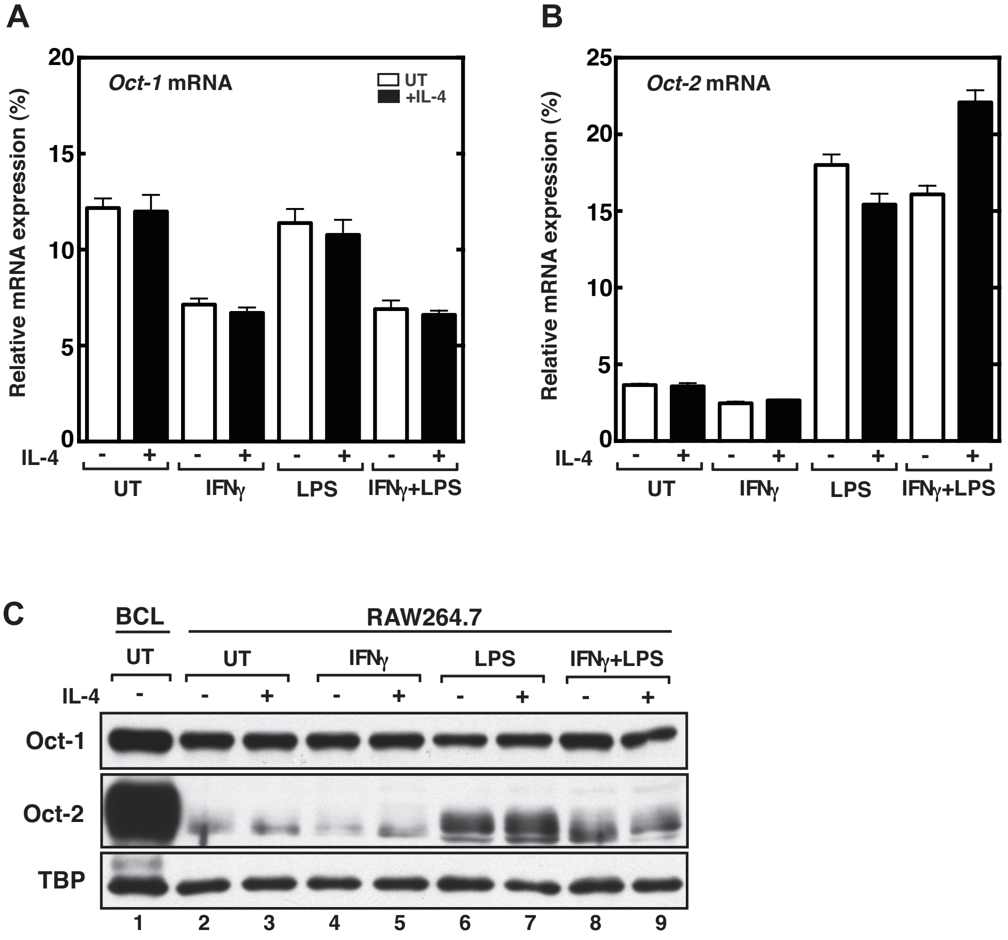

Supplemental Figure 2 Effect of IL-4 on Oct-1 and Oct-2 mRNA and protein expression in RAW264.7 cells stimulated with IFNγ and LPS

(A, B) RAW264.7 cells were treated with medium alone (untreated, UT) or IL-4 (10 ng/ml) for 30 min prior to stimulation with IFNγ (10 ng/ml) and/or LPS (100 ng/ml) for 8 hours before the preparation of total RNA and analysis of the *Oct-1* and *Oct-2* mRNA level by quantitative RT-PCR. The relative *Oct-1* and *Oct-2* mRNA expression levels are shown as percentages of their levels of expression in the mouse B cell leukemia cell line BCL1-B20 (BCL). Each column and bar represents the mean ± SEM of three independent experiments. (C) RAW264.7 cells were treated with cytokines and LPS, as described above, before the preparation of nuclear extracts. Twenty micrograms of the nuclear extract was analyzed by western blotting using anti-Oct-1, anti-Oct-2, and anti-TATA-binding protein (TBP) antibodies; nuclear extracts from BCL cells were used as a positive control. The data shown are representative of three independent experiments.

Supplemental Table 1

Gene	Sequence	Probe #	Accession number
Nos2	Forward 5’-ctttgccacggacgagac-3’	13	NM_010927
	Reverse 5’-tcattgtactctgagggctgac-3’		
Stat6	Forward 5’-tctccacgagcttcacattg-3’	42	NM_009284
	Reverse 5’-gaccaccaagggcagagac-3’		
Oct-1	Forward 5’-catggcaccctcacagttt-3’	67	NM_198933.2
	Reverse 5’-ctgagagcactgcccagagt-3’		
Oct-2	Forward 5’-gctgggcttcctacacagc-3’	25	NM_001163556
	Reverse 5’-aaggtgcgagcaaactgttc-3’		

Supplemental Table 2

Primer	Sequence	Restriction site
NOS -996 forward	5'-GCGAAG*ACGCGT*GGACCCCTGGCAGATGTGC-3'	*MluI*
NOS +104 reverse (P4)	5'-AGAAGT*AGATCT*AACAGCTCAGTCCCTTCA-3'	*BglII*
NOS -143 forward	5'-GGATACACCA*ACGCGT*GGGCCCATCAAGCACACAGAC-3'	*MluI*
NOS -86 forward	5'-GCACAC*ACGCGT*GGGGACTCTCCCTTTG-3'	*MluI*
NOS -62 forward	5'-TTGGGA*ACGCGT*ATGCAAAATAGCTCTG-3'	*MluI*
NOS -17 forward	5'-TAAATA*ACGCGT*GGCTGCTGCCAGGGTC-3'	*MluI*
mκB forward (P3)	5'-CCAACTGGCCAC*ATCGAT*TTTGGGAACAGTTATGCAAA-3'	*ClaI*
	NOS κB -85-GGGACTCTCC--76	
mκB reverse (P2)	5'-CCCAAA*ATCGAT*GTCGCCAGTTGGGTGTGCAAGTTAG-3'	*ClaI*
mOCT forward (P3)	5'-TTGGGA*ACGCGTCGTACG*AATAGCTCTGCAGAGCC-3'	*MluI, BsiWI*
	NOS OCT -61-ATGCAAAA--64	
mOCT reverse (P2)	5'-GCAGAGCTATT*CGTACG*AACTGTTCCCAAA-3'	*BsiWI*
Primer 1 forward (P1)	5'-GTGAGTCCCAGTTTTGAAGTGACTACGTGCTGCCTA-3'	

The restriction enzyme sites are indicated with italics. The underlined sequences represent the mutant κB and OCT sites. The wild-type nucleotide sequences of the κB and OCT sites are also shown.
